# Effect of Time-Restricted Eating Versus Daily Calorie Restriction on Mood and Quality of Life in Adults with Type 2 Diabetes

**DOI:** 10.3390/nu17172757

**Published:** 2025-08-26

**Authors:** Vasiliki Pavlou, Shuhao Lin, Sofia Cienfuegos, Mark Ezpeleta, Mary-Claire Runchey, Sarah Corapi, Krista A. Varady

**Affiliations:** 1 Department of Kinesiology and Nutrition, University of Illinois Chicago, Chicago, IL 60612, USA; pavlou2@uic.edu (V.P.); lin.shuhao@mayo.edu (S.L.); scienf2@uic.edu (S.C.); mrunch2@uic.edu (M.-C.R.); scorap2@uic.edu (S.C.); 2 Division of Endocrinology, Metabolism and Diabetes, University of Colorado School of Medicine, Aurora, CO 80045, USA; mezpel2@uic.edu

**Keywords:** time-restricted eating, intermittent fasting, calorie restriction, type 2 diabetes, mood, quality of life, depression, weight loss, obesity

## Abstract

Background/Objectives: This secondary analysis aimed to compare the effects of time-restricted eating (TRE) versus calorie restriction (CR) and controls on mood and quality of life in adults with type 2 diabetes (T2D). Methods: Adults with T2D (*n* = 69) were randomly assigned to one of three interventions for 6 months: 8 h TRE (eating only between 12 and 8 pm daily); CR (25% energy restriction daily); or a no-intervention control group. At baseline and 6 months, mood was assessed using the Beck Depression Inventory-II (BDI-II) and the Profile of Mood States (POMS) questionnaires, while quality of life was assessed using the Rand 36-Item Short Form (SF-36). Results: Body weight significantly decreased in the TRE group (−3.38%; 95% CI, −6.04 to −0.71%, *p* = 0.008), but not in the CR group (−1.80%, 95% CI, −4.50 to 0.91%, *p* = 0.32) versus controls by month 6. Fat mass, lean mass, and visceral fat mass remained unchanged in TRE and CR groups, versus controls, from baseline to month 6. No changes were observed in depression scores (BDI-II), total mood disturbance, or any POMS subscales (tension, depression, anger, fatigue, confusion, or vigor) in either the TRE or CR groups compared to controls. Similarly, there were no significant changes in the quality-of-life SF-36 constructs of vitality, bodily pain, mental health, and general physical health in the TRE or CR group versus controls. By month 6, there were no associations between changes in body weight, quality of life, and mood outcomes in any group. Conclusions: In conclusion, our findings suggest that TRE and CR do not have any effect on mood or quality of life in adults with T2D, relative to controls. However, the participants’ baseline mood and quality of life were generally within healthy ranges, and only minimal weight loss was achieved (3.5%, TRE only), which may explain the lack of observed effects.

## 1. Introduction

Diabetes is a chronic condition, and the goals of diabetes care include optimizing glycemic control, preventing complications, and supporting patients in maintaining or improving quality of life [1]. Individuals with type 2 diabetes (T2D) are twice as likely to experience depression compared to those without T2D, and successfully treating depression has been linked to improvements in glycemic control [2]. Furthermore, among those with T2D, a longer duration of the disease and comorbid depression are both associated with poorer quality of life [3].

While weight loss has been associated with improvements in quality of life and mood in adults with obesity [4,5], limited research has been conducted in those with T2D. In the Look AHEAD trial, an 8% reduction in body weight among those with type 2 diabetes was associated with a lower incidence of depressive symptoms, as well as improvements in the physical health component of quality of life [6]. Similarly, the DiRECT trial [7] found significant improvements in quality-of-life measures in participants with T2D who lost 10% of their body weight.

Recently, time-restricted eating (TRE) has been explored as an alternative to calorie restriction (CR) for weight loss in people with T2D. TRE involves consuming all daily calories within a defined window of 4 to 10 h, often resulting in an unintended reduction in caloric intake by 200–500 kcal, and subsequent reduction in body weight [8]. In a recent study, our group demonstrated that an 8-h TRE intervention led to a significant reduction in body weight of approximately 3.5%, relative to controls, in adults with T2D [9].

Despite these findings, it remains unclear how TRE versus CR impacts mood and quality of life in adults with T2D, a population at higher risk for depression and reduced quality of life. To date, only two studies [10,11] have examined the effect of TRE on mood and quality of life in individuals with T2D, and neither directly compared TRE and CR. The American Diabetes Association [12] note a growing interest in intermittent fasting, which highlights the need for more research in this population.

Therefore, this secondary analysis aims to compare the effect of 6 months of TRE versus CR and controls on mood and quality of life in adults with type 2 diabetes. We hypothesized that 8-h TRE would result in greater improvements in mood and quality of life, compared with the CR and control groups, due to the greater reductions in body weight in the TRE group.

## 2. Methods

### 2.1. Trial Participants

We conducted a secondary analysis of a previously published 6-month randomized controlled trial [9], which compared the effects of 8 h TRE with daily CR on body weight and glycemic control in adults with T2D [9]. Briefly, eligible participants were adults with previously diagnosed T2D, aged 18 to 80 years, with a baseline HbA1c between 6.5% and 11.0%, and a BMI of 30 to 50 kg/m^2^. Key exclusion criteria included greater than 4% weight change in the three months before enrollment, an eating window of less than 10 h per day, pregnant women, a history of eating disorders, night shift workers, or current smokers. Approval for the study protocol was obtained from the Office for the Protection of Research Subjects at the University of Illinois Chicago on 12 November 2024, and written informed consent was provided by all participants. The trial was registered on ClinicalTrials.gov (NCT05225337) on 25 January 2022.

### 2.2. Intervention Groups

Participants were randomized to one of three groups: TRE, CR, or control, for 6 months. Participants in the TRE group were instructed to consume food ad libitum between 12:00 PM and 8:00 PM, and abstain from eating from 8:00 PM to 12:00 PM the following day. Participants in the TRE group were not required to track their food or calorie intake during the 8-h eating window. Throughout the remaining 16-h fasting period, they were permitted to consume water and non-caloric beverages, such as plain coffee, tea, and up to two diet sodas per day. Participants in the CR group were instructed to reduce their caloric intake by 25% each day. Individual caloric requirements were estimated for each participant using the Mifflin equation [13], and adjusted by the applicable activity factor. CR subjects met with a dietitian at the start of the intervention to create personalized meal plans aligned with their dietary preferences. CR participants were required to use MyFitnessPal daily to track food intake. The control group was instructed to maintain their usual eating and exercise patterns throughout the 6 months of the study. Participants in the control group visited the lab at the same frequency as the intervention groups.

A study dietitian met with all participants weekly from baseline through month 3, via telephone or Zoom, followed by biweekly meetings from month 3 to month 6. Body weight, medication changes, and adverse events were documented during these visits. Only the TRE and CR groups received nutrition education based on the American Diabetes Association guidelines [12]. This included guidance on adhering to the intervention, as well as simple recommendations such as swapping refined grains for whole grains, including a protein and a vegetable at every meal, and avoiding sugar-sweetened beverages. All participants were asked to maintain their usual physical activity levels during the study.

### 2.3. Outcome Measures

All measures were collected at baseline and at month 6 [9]. Body weight was recorded using a digital scale at the research center, with participants wearing light clothing and after an overnight fast. Body composition, including fat mass, lean mass, and visceral fat mass, was measured using dual-energy x-ray absorptiometry (iDXA, GE HealthCare, Madison, WI, USA). To measure adherence, the TRE group was asked to log the time they began and stopped eating every day. Participants were marked as “adherent” if they indicated that they started and stopped eating within ±1 h of the prescribed eating window. The CR group was considered adherent if their caloric intake was within 200 calories of their assigned goals. This was determined via a 7-day food log assigned to all groups at baseline, month 3, and month 6.

Mood was measured using the Beck Depression Inventory II (BDI-II) [14] and the Profile of Mood States (POMS) [15]. BDI-II scores range from 0 to 63, with higher scores indicating more severe depression: 0–13 represents minimal or no depression, 14–19 mild depression, 20–28 moderate depression, and 29–63 severe depression. POMS includes six subscales with the following score ranges: tension (0–36), depression (0–60), anger (0–48), fatigue (0–28), vigor (0–32), and confusion (0–28). On the vigor subscale, higher scores indicate positive mood or emotional well-being, whereas lower scores on the other subscales suggest better mood states. The total mood disturbance score, ranging from −32 to 200, is calculated by adding the scores of the negative subscales (anger, depression, tension, confusion, and fatigue) and subtracting the vigor score, with higher total scores reflecting greater mood disturbances.

Quality of life was assessed using the Rand 36-Item Short Form (SF-36) [16], with scores ranging from 0 to 100 and higher values indicating a better perceived health. This survey includes four subscales: vitality and mental health, which assess the mental components of quality of life, and bodily pain and general physical health, which measure the physical aspects. To reduce time burden during lab visits, all questionnaires were completed remotely via REDCap (version 12.0.33 hosted at University of Illinois Chicago) prior to participants’ visits for measurements.

### 2.4. Statistical Analysis

Unless otherwise noted, all data are presented as mean (95% CI) and were analyzed for completers only (69 participants who completed the 6-month trial). Statistical significance was defined as a two-tailed *p*-value less than 0.05. We conducted a one-way analysis of covariance (ANCOVA) using baseline as the covariate. Pearson correlations were performed to assess the relationships between changes in body weight, mood, and quality of life. All data were analyzed using SPSS software (version 27, SPSS Inc., Chicago, IL, USA).

## 3. Results

### 3.1. Baseline Characteristics

**Figure 1:**
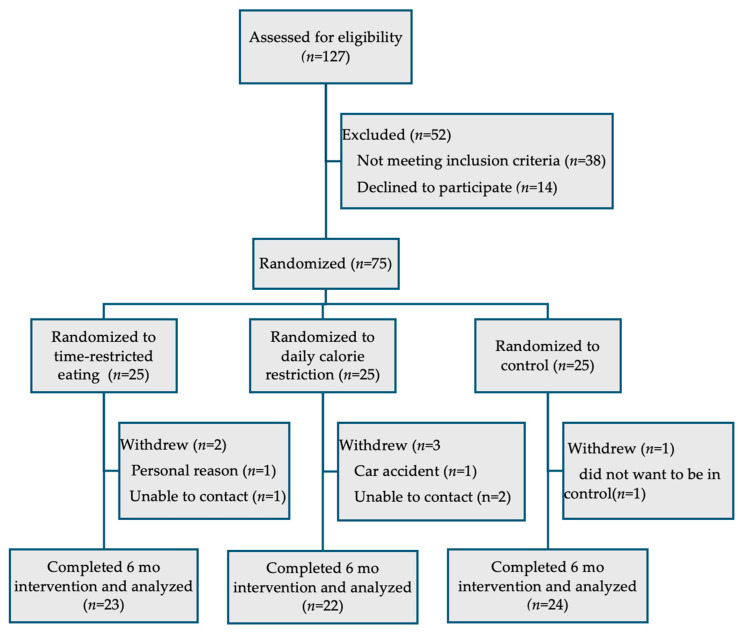
Participant flowchart.

As previously reported, 75 participants were recruited out of 127 screened (Figure 1) [9]. Six participants were lost to follow-up by month 6, including two from the TRE group, three from the CR group and one from the control group. Reasons for withdrawal included inability to contact, motor vehicle crash, not wanting to be in the control group, and personal reasons. For the present analysis, only the 69 participants who completed the study were included. Of the participants, two in the TRE group and one in the control group did not complete the mood and quality of life questionnaires. Baseline characteristics for the completers are presented in Table 1.

### 3.2. Body Weight and Body Composition

Body weight and composition changes are reported in Table 2. Body weight significantly decreased in the TRE group (−3.38%; 95% CI, −6.04 to −0.71%, *p* = 0.008), but not in the CR group (−1.80%, 95% CI, −4.50 to 0.91%, *p* = 0.32) versus controls, by month 6. There were no significant changes in fat mass, lean mass, and visceral fat mass in TRE and CR completers, versus controls, from baseline to month 6.

### 3.3. Depression Scores and Total Mood Disturbance

**Figure 2:**
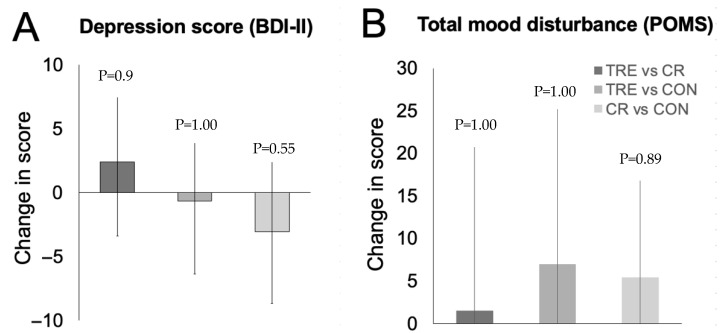
Change in mood scores between TRE, CR, and CON by month 6. Data were included for 69 participants (completers only). Error bars indicate 95% confidence intervals for each parameter from baseline by diet group. (**A**) Change in depression score (measured with the Beck Depression Inventory II (BDI-II) survey). (**B**) Change in total mood disturbance score (measured with Profile of Mood States survey (POMS)). Abbreviations: BDI-II: Beck Depression Inventory II; CON: control; CR: calorie restriction; POMS: Profile of Mood States survey; TRE: time-restricted eating.

Changes in depression score and total mood disturbance are reported in Table 2 and Figure 2. At baseline, the BDI-II survey indicated that participants in the TRE, CR, and control groups had mild depression. There were no significant changes in BDI-II depression scores in either the TRE or CR groups compared to controls at month 6. At baseline, scores on the POMS subscales for tension, depression, anger, fatigue, and confusion were low across all groups, while the vigor subscale—reflecting good mood—was high. Additionally, total mood disturbance scores were low at baseline, suggesting that participants across groups generally reported positive mood at the start of the trial. There were no significant changes in the total mood disturbance score or any of the POMS subscales in the TRE or CR groups versus controls by month 6.

### 3.4. Quality of Life

**Figure 3:**
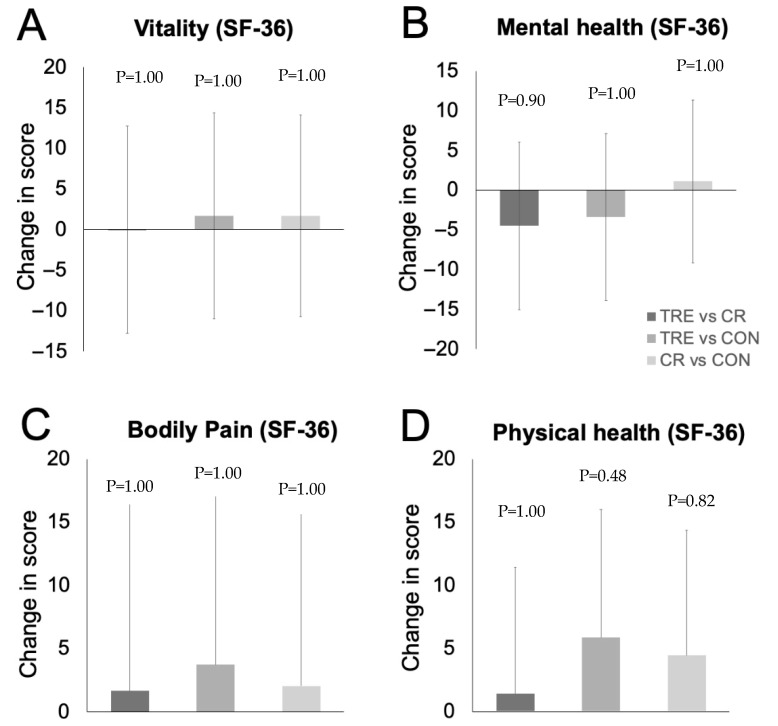
Change in quality-of-life constructs between TRE, CR, and CON by month 6. Data were included for 69 participants (completers only). Error bars indicate 95% confidence intervals for each parameter from baseline by diet group. (**A**) Change in vitality score (measured with the SF-36 survey). (**B**) Change in mental health score (measured with the SF-36 survey). (**C**) Change in bodily pain score (measured with the SF-36 survey). (**D**) Change in general physical health score (measured with the SF-36 survey). Abbreviations. CON: control; CR: calorie restriction; SF-36: Rand 36-Item Short Form survey; TRE: time-restricted eating.

Changes in quality of life are reported in Table 2 and Figure 3. The SF-36 constructs of vitality, mental health, bodily pain, and general physical health were all moderately high in all groups at baseline, indicating good quality of life. At month 6, there were no significant changes between the TRE or CR groups and controls in measures of mental health, bodily pain, or overall physical health. Changes in body weight were not associated with mood or quality of life in the TRE, CR, or control group by month 6.

## 4. Discussion

This study is, to our knowledge, the first to compare the effects of TRE vs. CR on mood and quality of life in individuals with T2D. Our findings suggest that neither TRE nor CR produced significant changes in mood or quality of life over a 6-month period when compared to a non-intervention control group.

At baseline, participants in our study had mild depressive symptoms and generally good mood and quality of life. BDI-II scores were 12 in the TRE group and 15 in the CR and control groups, reflecting mild depression, while POMS scores for tension, depression, anger, fatigue, and confusion were low, and vigor scores were moderate, suggesting that our participants entered the study with relatively stable mood profiles. SF-36 scores for vitality (50–57), mental health (72–79), and physical health (47–56) suggested good mental health and average vitality, but slightly lower physical health at baseline. Compared with large lifestyle trials such as Look AHEAD [17] and the Diabetes Prevention Program [18], our participants’ baseline mood and quality of life were broadly similar, though our groups showed mild depression, whereas the aforementioned trials reported normal BDI-II scores. In the Diabetes Prevention Program [18], baseline SF-36 physical component summary, mental component summary, and BDI-II scores were 50.3 ± 7.1, 54.0 ± 7.5, and 4.6 ± 4.6, respectively. The Look AHEAD trial [17] reported baseline physical component summary of 47.9 ± 7.9, mental component summary of 54.0 ± 8.1, and a mean BDI-II score of 5.7 ± 5.0. Physical and mental component summary scores from the SF-36 capture overall functioning and provide useful context, but are not directly comparable to our domain-level SF-36 scores.

Adults with diabetes are more likely to experience depression compared to the general population [2], which is associated with treatment nonadherence [19] and negative effects on their glycemic control [20]. We found that after 6 months, 8-h TRE produced no significant changes in mood or depressive symptoms. To our knowledge, no prior studies have specifically examined the effects of TRE on mood or depressive symptoms in individuals with type 2 diabetes. Our findings contrast with the Look AHEAD trial [6], in which an intensive lifestyle intervention leading to 8% weight loss significantly reduced the incidence of mild or greater depressive symptoms in participants with type 2 diabetes. Similarly, a study comparing low-fat and low-carbohydrate diets in individuals with T2D found that both diets resulted in weight loss of approximately 9% and improvements in mood (measured by BDI) and depressive symptoms (measured by POMS); these improvements were significantly correlated with a decrease in both body weight and glycated hemoglobin [21]. The absence of mood-related changes in our study may reflect the relatively modest weight loss achieved with an 8-h TRE window, which may be insufficient to produce improvements in mood and depressive symptoms in individuals with T2D. Importantly, while we did not observe improvements in mood, our findings suggest that TRE does not have negative effects on these outcomes in individuals with type 2 diabetes—a group already at elevated risk for depression. This is a key consideration for clinicians, as it provides reassurance that TRE may be recommended for its potential weight and glycemic benefits without concern for worsening mood.

With one in four individuals with diabetes experiencing elevated depressive symptoms or being diagnosed with depressive disorders, it is recommended that clinicians routinely screen for depressive mood in this population [22]. However, the relationship between diabetes and depression is complex and can be influenced by many factors. Depression can result from the challenge of managing progressive chronic conditions like diabetes; yet, prospective studies also suggest that depression independently increases the risk of developing T2D [2].

Among those with T2D, depression is further associated with reduced quality of life [3]. Given the greater incidence of depression among individuals with T2D and its association with poorer quality of life and treatment adherence, understanding how lifestyle interventions like TRE affect mood is clinically important. There is some concern that TRE could negatively affect quality of life if it interferes with an individual’s social life. However, a systematic review found that TRE had either a neutral or positive effect on quality of life across various populations [23]. Our study adds to this body of evidence by showing no change in quality of life among participants with T2D who followed a TRE regimen. These findings suggest that for individuals who wish to try TRE for weight or glycemic benefits, their quality of life is unlikely to be negatively affected by this dietary approach. However, the absence of improvements in quality of life may reflect the modest degree of weight loss achieved by the TRE regimen [24]. This is supported by two large trials [6,7] in individuals with type 2 diabetes that have been shown to improve quality of life following caloric restriction. The Look AHEAD trial [6] reported an improvement in the physical functioning domain of quality of life (change: 1.65 [SD7.94]) in participants in the intensive lifestyle intervention group, where the average weight loss was 8%. Similarly, in the DiRECT trial [7], participants in the intervention group achieved a mean weight loss of 10% and a seven-point improvement in quality of life, as measured by the EuroQol 5-Dimensions Visual Analogue Scale (EQ-5D VAS), after 12 months.

At present, only two studies have assessed the impact of TRE on quality of life in people with T2D. A 12-week study by Chet et al. [10] found that 10-h TRE significantly reduced body weight by 4% and improved quality of life, as assessed by the 12-item Short Form survey (SF-12), compared to the control group. The SF-12 is a shortened version of the SF-36 that captures the same physical and mental health domains but with fewer questions. In contrast, a pre-post study by Parr et al. [11] reported no change in quality of life after 4 weeks of 9-h TRE. This shorter duration study, compared with the 6-month intervention in the present study, and the absence of weight loss may have been insufficient to detect changes in quality of life.

In our study, there was no difference in body weight between the TRE and CR groups, which may help explain why the two interventions did not produce different changes in mood or quality of life. A previous one-year study [25] from our lab directly comparing TRE and CR in people with obesity supports our findings. Both interventions produced similar energy restriction (~400 kcal/day) and comparable weight loss (~5%) between groups, and neither led to changes in mood or quality of life. These results reinforce the idea that TRE and CR are essentially different approaches to achieving calorie reduction.

Lastly, we found no associations between changes in body weight, mood, or quality of life in any group. In contrast, in the Diabetes Prevention Program, Ackermann et al. [18] quantified the amount of weight loss necessary to see improvements in QOL regardless of treatment group (metformin, lifestyle intervention, or placebo) and reported that every 5 kg weight loss was associated with an increase of 0.64 in the SF-36 physical component and an increase of 0.28 in the SF-36 mental component. In terms of mood, a lifestyle intervention study [26] found significant improvements in BDI-II scores after 12 months of 4.2% (SD 6.1%) weight loss, and a 1-unit decrease in BDI-II score was associated with a further −0.4% decrease in body weight. These findings suggest that the modest weight loss (3.5%) in our study may have been insufficient to detect associations between weight change and mood or quality of life.

Our study has several limitations. First, the sample size was small (*n* = 69), likely limiting our statistical power to detect significant differences in secondary outcomes. Second, mood, quality of life, and dietary adherence were assessed using self-reported questionnaires, which are subject to response bias. Third, our study population exhibited low depression and moderately high quality of life scores at baseline, which may have hindered our ability to detect improvements in these variables. Finally, no adjustment was made for multiple comparisons in the analysis of secondary outcomes. Future studies could build on these findings by including larger sample sizes, targeting participants with lower baseline mood or quality of life, and using clinician-administered assessments to more accurately capture changes in mood and quality of life.

## 5. Conclusions

In conclusion, our findings suggest that TRE and CR do not have any effect on mood or quality of life in adults with obesity and T2D over 6 months, relative to controls. However, our participants had healthy mood and quality of life scores at baseline and experienced only modest weight loss (3.5%), which may explain why no significant changes were observed. These findings should be interpreted with caution due to the small sample size, which may have limited our ability to detect changes in mood or quality of life. Future studies with larger sample sizes and longer follow-up periods are needed to confirm these findings.

## Figures and Tables

**Table 1:** Baseline characteristics.

Variables	Time-Restricted Eating (TRE)	Calorie Restriction (CR)	Control (CON)
*n*	23	22	24
Age (y)	57 (13)	56 (12)	54 (11)
Sex (no. female/male)	17/6	17/5	17/7
Diabetes duration (y)	16 (11)	15 (8)	13 (9)
Body weight and composition			
Body weight (kg)	106 (26)	102 (17)	108 (22)
Fat mass (kg)	47 (15)	48 (11)	49 (17)
Lean mass (kg)	54 (11)	50 (9)	54 (10)
Visceral fat mass (kg)	1.9 (0.6)	2.4 (1.0)	2.2 (1)
BMI (kg/m^2^)	39 (9)	38 (5)	40 (7)
Mood			
BDI-II, Depression score	12 (8)	15 (9)	15 (8)
POMS, Tension	2 (4)	4 (4)	4 (5)
POMS, Depression	2 (4)	3 (5)	4 (4)
POMS, Anger	2 (3)	4 (5)	3 (3)
POMS, Fatigue	3 (3)	4 (3)	4 (3)
POMS, Vigor	8 (4)	7 (3)	7 (4)
POMS, Confusion	2 (2)	3 (3)	4 (4)
POMS, Total mood disturbance score	−13 (18)	−4 (21)	−1 (22)
Quality of Life			
SF-36, Vitality	57 (16)	52 (20)	50 (20)
SF-36, Mental health	79 (15)	74 (18)	72 (14)
SF-36, Bodily pain	76 (20)	58 (22)	61 (26)
SF-36, General physical health	56 (15)	54 (20)	47 (18)

Data are expressed as mean (SD). Abbreviations: BMI: body mass index; BDI-II: Beck Depression Inventory II; CR: calorie restriction; POMS: Profile of Mood States; SF-36: Rand 36-Item Short Form; TRE: time-restricted eating.

**Table 2:** Change in body weight, body composition, mood, and quality of life parameters over 6 months.

Variables	Change from Baseline to Month 6 (95% CI)			Difference Between Groups by Month 6 (95% CI)		
	Time-Restricted Eating (TRE)	Daily Calorie Restriction (CR)	Control (CON)	TRE vs. CR	TRE vs. CON	CR vs. CON
Body weight and composition						
Body weight (%)	−4.16 (−5.70, −2.61)	−2.58 (−4.16, −0.99)	−0.78 (−2.30, 0.74)	−1.58(−4.31, 1.15), *p* = 0.48	−3.38 (−6.04, −0.71), *p* = 0.008 *	−1.80 (−4.50, 0.91), *p* = 0.32
Body weight (kg)	−4.39 (−6.06, −2.73)	−2.71 (−4.41, −1.00)	−1.10 (−2.74, 0.53)	−1.69 (−4.62, 1.25), *p* = 0.49	−3.29 (−6.15, −0.42), *p* = 0.02 *	−1.60 (−4.52, 1.31), *p* = 0.54
Fat mass (kg)	−2.35 (−3.73, −0.98)	−1.92 (−3.32, −0.52)	−0.38 (−1.72, 0.96)	−0.44 (−2.85, 1.98), *p* = 1.00	−1.97 (−4.34, 0.40), *p* = 0.13	−1.54 (−3.93, 0.85), *p* = 0.36
Lean mass (kg)	−1.29 (−2.23, −0.35)	−0.90 (−1.87, 0.63)	−0.74 (−1.66, 0.18)	−0.39 (−2.05, 1.28), *p* = 1.00	−0.55 (−2.16, 1.06), *p* = 1.00	−0.16 (−1.81, 1.49), *p* = 1.00
Visceral fat mass (kg)	−0.16 (−0.32, 0.02)	−0.18 (−0.32, −0.01)	−0.03 (−0.18, 0.12)	0.01 (−0.27, 0.28), *p* = 1.00	−0.13 (−0.40, 0.15), *p* = 0.77	−0.14 (−0.40, 0.13), *p* = 0.64
Mood						
BDI-II, Depression score	−2.90 (−6.25, 0.47)	−5.30 (−8.57, −2.04)	−2.24 (−5.43, 0.95)	2.41 (−3.39, 8.21), *p* = 0.93	−0.65 (−6.37, 5.06), *p* = 1.00	−3.06 (−8.67, 2.54), *p* = 0.55
POMS, Tension	0.90 (−1.42, 1.60)	0.43 (−1.02, 1.87)	−0.62 (−2.04, 0.80)	−0.34 (−2.93, 2.26), *p* = 1.00	0.71 (−1.86, 3.28), *p* = 1.00	1.05 (−1.44, 3.53), *p* = 0.91
POMS, Depression	−1.40 (−2.95, 0.15)	−0.34 (−1.84, 1.16)	−0.48 (−1.96, 1.00)	−1.06 (−3.72, 1.60), *p* = 1.08	−0.92 (−3.57, 1.73), *p* = 1.00	0.14 (−2.45, 2.73), *p* = 1.00
POMS, Anger	−1.15 (−2.65, 0.34)	0.07 (−1.38, 1.51)	−0.38 (−1.78, 1.04)	−1.22 (−3.82, 1.38), *p* = 0.76	−0.80 (−3.32, 1.73), *p* = 1.00	0.42 (−2.05, 2.90), *p* = 1.00
POMS, Fatigue	−0.13 (−1.38, 1.13)	0.14 (−1.08, 1.35)	−0.63 (−1.82, 0.57)	−0.26 (−2.42, 1.89), *p* = 1.00	0.50 (−1.64, 2.64), *p* = 1.00	0.76 (−1.33, 2.85), *p* = 1.00
POMS, Vigor (good mood)	1.04 (−0.80, 2.88)	0.54 (−1.24, 2.33)	1.10 (−0.65, 2.85)	−0.50 (−2.68, 3.66), *p* = 1.00	−0.06, (−3.20, 3.08), *p* = 1.00	−0.56 (−3.62, 2.51), *p* = 1.00
POMS, Confusion	−0.45 (−1.45, 0.56)	0.31 (−0.66, 1.28)	−1.29 (−2.25, −0.03)	−0.76 (−2.47, 0.96), *p* = 0.84	0.84 (−0.90, 2.58), *p* = 0.72	1.60 (−0.09, 3.29), *p* = 0.07
POMS, Total mood disturbance score	−0.13 (−10.94, 10.69)	−1.65 (−9.92, 6.62)	−7.09 (−15.03, 0.857)	1.53 (−17.75, 20.80), *p* = 1.00	6.96 (−11.85, 25.77), *p* = 1.00	5.44 (−7.18, 18.06), *p* = 0.89
Quality of Life						
SF-36, Vitality	5.32 (−2.12, 12.76)	5.32 (−1.90, 12.53)	3.61 (−3.47, 10.70)	−0.001 (−12.79, 12.79), *p* = 1.00	1.71 (−11.01, 14.43), *p* = 1.00	1.71 (−10.72, 14.13), *p* = 1.00
SF-36, Mental health	−0.65 (−6.79, 5.50)	3.84 (−2.10, 9.78)	2.74 (−3.10, 8.58)	−4.49 (−15.03, 6.06), *p* = 0.90	−3.39 (−13.89, 7.11), *p* = 1.00	1.10 (−9.13, 11.33), *p* = 1.00
SF-36, Bodily pain	2.47 (−6.21, 11.15)	0.80 (−7.51, 9.12)	−1.26 (−9.30, 6.77)	1.67 (−13.47, 16.80), *p* = 1.00	3.73 (−10.98, 18.45), *p* = 1.00	2.07 (−12.05, 16.19), *p* = 1.00
SF-36, General physical health	5.93 (0.05, 11.80)	4.50 (−1.19, 10.20)	0.06 (−5.58, 5.71)	1.42 (−8.61, 11.46), *p* = 1.00	5.86 (−4.28, 16.00), *p* = 0.48	4.44 (−5.46, 14.34), *p* = 0.82

Data were included for 69 participants (completers only). Error bars indicate 95% confidence intervals for each parameter from baseline by diet group. Abbreviations: BDI-II: Beck Depression Inventory II; CON: control; CR: calorie restriction; POMS: Profile of Mood States; SF-36: Rand 36-Item Short Form; TRE: time-restricted eating. * Indicates statistical significance between groups using *p* < 0.05.

## Data Availability

The original contributions presented in the study are included in the article; further inquiries can be directed to the corresponding authors.
